# The Effect of Light and Dark Treatment on the Production of Rosmarinic Acid and Biological Activities in *Perilla frutescens* Microgreens

**DOI:** 10.3390/plants12081613

**Published:** 2023-04-10

**Authors:** Seom Lee, Hyeon Ji Yeo, Sang Yeob Lee, Su Ryang Kim, Sang Un Park, Chang Ha Park

**Affiliations:** 1 Department of Biological Sciences, Keimyung University, 1095 Dalgubeol-daero, Daegu 42601, Republic of Korea; 2 Biological Resource Center, Korea Research Institute of Bioscience and Biotechnology (KRIBB), 181 Ipsin-gil, Jeongeup 56212, Republic of Korea; 3 Department of Crop Science, Chungnam National University, 99 Daehak-ro, Daejeon 34134, Republic of Korea; 4 Department of Smart Agriculture Systems, Chungnam National University, 99 Daehak-ro, Daejeon 34134, Republic of Korea

**Keywords:** *Perilla frutescens*, microgreens, dark and light treatments, rosmarinic acid, antioxidant and antibacterial effect

## Abstract

This study aimed to investigate the effect of light [a long-day photoperiod (16 h light/8 h dark cycle)] and dark treatment on the production of rosmarinic acid in *P*. *frutescens* microgreens and to determine its antioxidant and antibacterial activities. Microgreens of *P*. *frutescens* were grown under light and dark conditions and harvested after 10, 15, 20, and 25 days of each treatment. Although dry weight values of microgreens gradually increased from 10 to 25 days of both treatments, the microgreens grown under light treatment possessed slightly higher levels of dry weight than those grown in the dark. Rosmarinic acid and total phenolic content (TPC) were also analyzed using high-performance liquid chromatography (HPLC) and Folin–Ciocalteu assay. The accumulation patterns of rosmarinic acid and TPC gradually increased and decreased, respectively, in *P*. *frutescens* microgreens grown in continuous darkness. The highest accumulation was observed in microgreens grown for 20 days. However, rosmarinic acid and TPC values were not significantly different in microgreens grown under light conditions. According to the 2,2-diphenyl-1-picrylhydrazyl (DPPH) radical inhibition assay, the extracts of *P*. *frutescens* microgreens were confirmed to be strong antioxidants, and their ability to scavenge DPPH radicals was positively correlated with the total phenolic content in the microgreens after 10, 15, 20, and 25 days of both treatments. Considering the relatively higher values of dry weight, rosmarinic acid, TPC, and DPPH assay, *P*. *frutescens* microgreens after 20 days of darkness and 20 days of light treatment, respectively, were selected for screening antibacterial activity using nine pathogens. Both microgreen extracts showed strong antibacterial activity against pathogens. In particular, the extracts of microgreens grown for 20 days under light treatment showed higher antimicrobial effects. Therefore, the light treatments for 20 days, as well as the darkness treatment for 20 days, were the best conditions for *P*. *frutescens* microgreen production because of their high levels of dry weight, phenolics, and biological activities.

## 1. Introduction

*Perilla frutescens*, also known as the Korean perilla or Dlggae, is a member of the Labiatae family distributed in China, Korea, and Japan. It is an annual herbaceous plant that has been used in medicinal plants and food [1]. Its seeds are commercially used for perilla seed oil production [2] and its leaves are used for perilla essential oil production, culinary garnishes, and seasoning [3,4]. Traditionally, *P*. *frutescens* has been used as a medicinal herb for the treatment of allergies, asthma, cough, depressive disorders, tumors, intoxication, fever, shivering, headache, and congested nose [4]. The use of *P*. *frutescens* as an herbal medicine may be due to its strong antioxidant [5], anticancer [6], antiallergic [7], and antibacterial [8] properties, which may be derived from secondary metabolites biosynthesized in *P*. *frutescens*. Hou et al. (2022) listed secondary metabolites (alkaloids, terpenes, phenolics, glycosides, and benzoxipen derivatives) found in the seeds, leaves, stems, and fruits of *P*. *frutescens* [2] and reported their biological functions. For example, Lee et al., 2021 identified caffeic acid, ferulic acid, and rosmarinic acid in this plant and reported a positive correlation between its antioxidant ability and the composition of phenolic acids (caffeic acid and rosmarinic acid) [5]. Rosmarinic acid and caffeic acid have been considered effective antioxidants against free radicals [9]. Rosmarinic acid, caffeic acid, luteolin, apigenin, methoxyflavanone, and alpha-linolenic acid present in *P*. *frutescens* were reported to have anti-allergic properties [10]. Rosmarinic acid of *P*. *frutescens* leaves has been shown to have antimicrobial activity against bacterial and fungal pathogens [11], and the activity of *P*. *frutescens* leaf extract against apoptosis in hepatic carcinoma (Hep-G2) cells has been shown to be due to rosmarinic acid, caffeic acid, luteolin, and triterpenes [12].

Rosmarinic acid is a naturally occurring caffeic acid ester commonly present in herbs (rosemary, Korean mint, sage, basil, oregano, marjoram, and lemon balm) belonging to the Labiatae family [13]. In plant rosmarinic acid biosynthesis, tyrosine and phenylalanine, which are amino acids, are the first precursors that can be converted into 4-hydroxyphenyllactic acid and 4-coumaroyl-CoA, respectively, by serial enzyme reactions. These two intermediates were condensed into 4-coumaroyl-4′-hydroxyphenyllactic acid by rosmarinic acid synthase (RAS). The resulting metabolite may be converted to rosmarinic acid by cytochrome P450-dependent monooxygenase (CYP98A14) [14]. Various biological functions, such as antioxidant, anti-allergenic, antidepressant, and antibacterial activities of rosmarinic acid, have been reported [15].

Microgreens, which are defined as plant seedlings older than sprouts but younger than baby greens, are characterized by high amounts of bioactive compounds [16]. Microgreens are often more nutrient- and phytochemical-dense than seeds or mature plants [17], and edible microgreens can aid human health because of their health benefits associated with plant secondary metabolites [16]. A recent study reported that the abundance of volatiles was higher during the microgreen stage of *P*. *frutescens* var. frutescens and *P*. *frutescens* var. *crispa* than in the adult stage (4 weeks stage) [18]. Furthermore, microgreens of herbs or medicinal plants, such as fenugreek [17,19] and Korean ginseng [17,20], exhibit high levels of health-maintaining phytochemicals and biological properties. Therefore, herbal or medicinal microgreens can be a source of functional foods that are beneficial to human health.

Microgreens can be produced under a long-day photoperiod or continuous darkness conditions, and these different conditions can affect the growth and production of phytochemicals in microgreens. Even if it is helpful to investigate optimal cultivation conditions for microgreen production, to date there have been no previous studies on the effects of light and dark treatment on rosmarinic acid accumulation and biological activities in *P*. *frutescens* microgreens. Thus, this study aimed to investigate the microgreen dry weight, production of rosmarinic acid, and antioxidant and antibacterial effects of *P*. *frutescens* microgreens grown under light and dark conditions for 10, 15, 20, and 25 days.

## 2. Results

### 2.1. P. frutescens Microgreen Production

**Figure 1:**
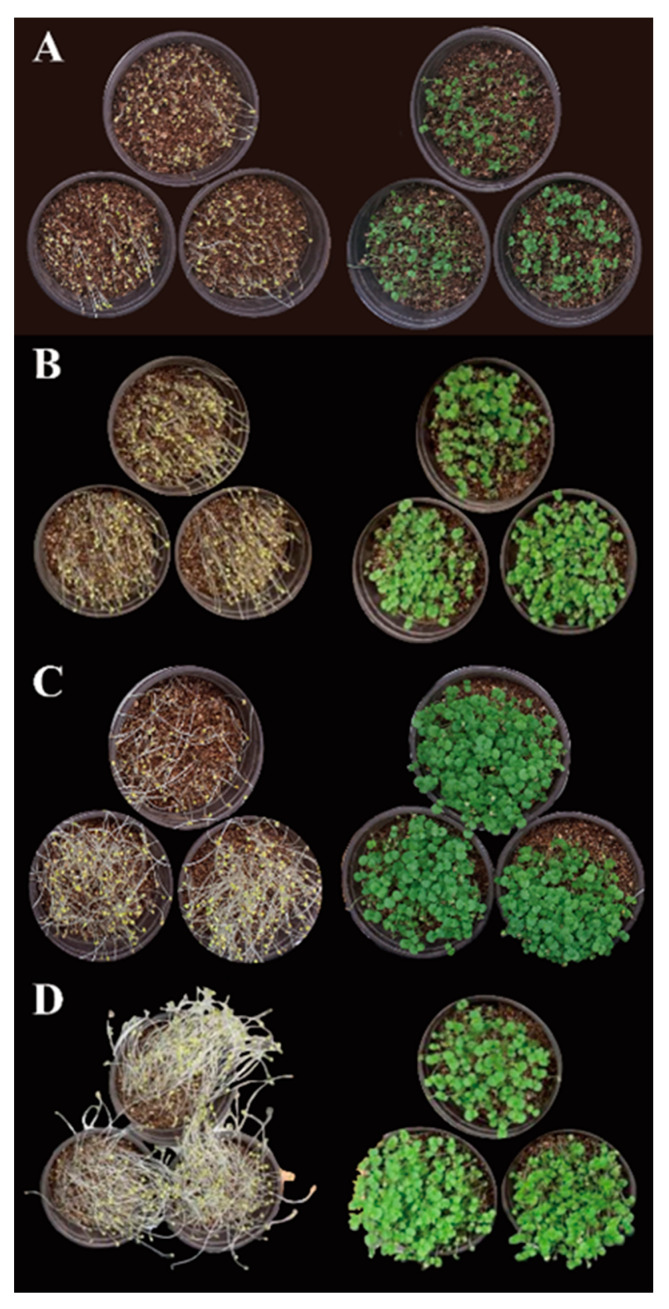
*P. frutescens* microgreens grown under long-day photoperiod or continuous darkness conditions. (**A**) Microgreens grown for 10 days under dark condition (**left**) and microgreens grown for 10 days under long-day photoperiod condition (**right**); (**B**) microgreens grown for 15 days under dark condition (**left**) and microgreens grown for 15 days under long-day photoperiod condition (**right**); (**C**) microgreens grown for 20 days under dark condition (**left**) and microgreens grown for 20 days under long-day photoperiod condition (**right**); and (**D**) microgreens grown for 25 days under dark condition (**left**) and microgreens grown for 25 days under long-day photoperiod condition (**right**).

*P*. *frutescens* microgreens were generated in pots containing vermiculites under light (long-photoperiod conditions) and continuous darkness treatments for 10, 15, 20, and 25 days. Microgreens grown in darkness exhibited yellow cotyledons and hypocotyl elongation. In contrast, the light treatment allowed the microgreens to reveal green cotyledons and emerged young leaves (Figure 1). As shown in Table 1, the dry weight values in the microgreens grown under both treatments gradually increased in a time-dependent manner, and the highest values were obtained at 20 and 25 days in both treatments. These findings suggest that light treatment for 20 or 25 days and darkness treatment for 20 or 25 days were suitable for the production of *P*. *frutescens* microgreens considering the dry weight values.

### 2.2. Rosmarinic Acid Production and Total Phenolic Contents in P. frutescens Microgreens

Table 2 shows the patterns of rosmarinic acid production in *P*. *frutescens* microgreens grown under different treatments. Rosmarinic acid values in *P*. *frutescens* microgreens grown under dark treatment ranged from 5.48 ± 4.46 (10 days) to 13.76 ± 0.33 mg/g DW (20 days), a difference of 2.51 times. The values gradually increased and then decreased from 10 to 25 days of dark treatment, and the highest value was obtained from *P*. *frutescens* microgreens grown for 20 days. However, rosmarinic acid values in *P*. *frutescens* microgreens were not significantly different during the duration of light treatment. Similarly, the total phenolic contents (TPC) were measured in *P*. *frutescens* microgreens grown under different treatments. TPC levels were not significantly different in *P*. *frutescens* microgreens grown under light treatment for 10, 15, 20, and 25 days. However, the TPC levels of *P*. *frutescens* microgreens grown under dark treatment gradually increased from day 10 to day 20 and then decreased (Table 3). Furthermore, the DDPH free radical inhibition activity was assessed with ethanol extracts of *P*. *frutescens* microgreens grown under different treatments (Table 4). Microgreens grown in both treatments showed strong antioxidant abilities. Specifically, the inhibition activities in *P*. *frutescens* microgreens grown for 10, 15, 20, and 25 days were not significantly different. In contrast, the activity gradually increased and decreased during continuous darkness treatment. These findings are consistent with the accumulation patterns of rosmarinic acid and TPC obtained from *P*. *frutescens* microgreens grown under both treatments. Therefore, the findings in Table 2 and Table 3 demonstrate that of the durations of continuous darkness and long-day photoperiod conditions, 20-day darkness and 20-day long-day photoperiod conditions are the best for the production of rosmarinic acid and TPC and its antioxidant activity.

### 2.3. In Vitro Antibacterial Properties of Methanol Extracts of P. frutescens Microgreens for 20 Days of Long-Day Photoperiod or Continuous Darkness Conditions

**Figure 2:**
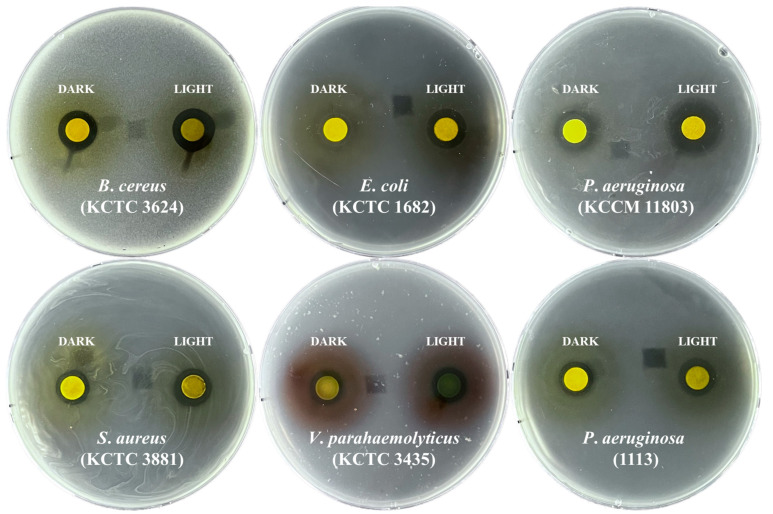
Representative images showing antibacterial activities of methanol extracts of *P*. *frutescens* microgreens for 20 days of long-day photoperiod and continuous darkness conditions.

Considering the dry weight values, rosmarinic acid production, total phenolic content, and antioxidant activity, *P*. *frutescens* microgreens subjected to 20 days of long-day photoperiod and dark treatment are the most suitable for antibacterial screening against pathogens (Figure 2). Bacterial growth was inhibited by methanol extracts of *P*. *frutescens* microgreens grown under both treatments on an agar medium containing *B*. *cereus* (KCTC 3624), *E*. *coli* (KCTC 1682), *P*. *aeruginosa* (KCCM 11803), *S*. *aureus* (KCTC 3881), *V*. *parahaemolyticus* (KCTC 2471), and *P*. *aeruginosa* (1113). However, growth was not inhibited in an agar medium containing *M*. *luteus* (KCTC 3063), *S*. *mutans* (KCTC 3065), or *S*. *paratyphi* C (KCCM 41577) (Table 4). Differences in antibacterial activities against eight normal pathogens and one multidrug-resistant pathogen were observed in the extracts of microgreens grown under both treatments. In particular, extracts of *P*. *frutescens* microgreens grown for 20 days with a long-day photoperiod showed slightly higher antibacterial effects against *B*. *cereus* (KCTC 3624), *E*. *coli* (KCTC 1682), *P*. *aeruginosa* (KCCM 11803), *S*. *aureus* (KCTC 3881), *V*. *parahaemolyticus* (KCTC 2471), and *P*. *aeruginosa* (1113).

## 3. Discussion

Plant microgreens are commonly produced using different photoperiods or sequential darkness. These growth conditions can cause differences in phenotype, growth, development, and metabolism because light is necessary for plant photosynthesis [21]. In this study, *P*. *frutescens* microgreens were successfully grown under light treatment (16 h/8 h light-dark) and continuous treatment for 10, 15, 20, and 25 days. *P*. *frutescens* microgreens grown in darkness possessed yellow cotyledons, and the microgreens grown in the light treatment had green cotyledons. This may have been due to the accumulation of chlorophyll and carotenoids. Previously, chlorophyll and carotenoid biosynthetic genes were upregulated when the plant seedlings were exposed to light [22], and lower levels of carotenoids and chlorophylls were confirmed in radish, soybean, mung bean, and pumpkin spouts compared with light treatment [23]. Despite these phenotypic differences, the growth patterns (dry weight) of the microgreens grown under both conditions were not significantly different. Furthermore, the accumulation patterns of phenolics (rosmarinic acid and total phenolic content) in *P*. *frutescens* microgreens were not significantly different during the long photoperiod. However, the production patterns of phenolics gradually increased and then decreased during sequential darkness. Twenty days of cultivation in the dark resulted in the highest production of rosmarinic acid and total phenolic content. Previous studies have reported that variations in the production of phenolic compounds in plant microgreens may be due to different plant species, germination stages, light sources, and photoperiods. For example, Mastropasqua et al. (2020) reported that the production of total phenolic content was not significantly different in radish, soybean, mung bean, and pumpkin spouts grown under a long photoperiod or in continuous darkness [23]. However, Siriparu et al. (2022) reported that total phenolic content in mung bean sprouts gradually increased during five days of continuous darkness and short photoperiod (12/12 h) conditions, and the sprouts grown in the dark showed higher levels of total phenolic content [24]. Furthermore, Nam et al. (2017) reported that buckwheat sprouts produced higher levels of total phenolics under blue light, followed by fluorescent light, red light, and darkness treatment [25]. Xiang et al. (2017) showed that gallic acid, chlorogenic acid, syringic acid, and hydroxycinnamic acid levels were not significantly different in sweet corn sprouts under light and dark conditions. However, ferulic acid levels were higher in sprouts treated with light [26]. Therefore, this study highlighted the establishment of optimal cultivation conditions and suggested that 20 days and 25 days of day-cultivation under long-photoperiod conditions were optimal, considering microgreen growth and phenolic compound accumulation, and 20 day-cultivation in continuous darkness was appropriate for the microgreen production of *P*. *frutescens*.

Rosmarinic acid was detected in *P*. *frutescens* microgreens (mainly root, hypocotyl, and cotyledons) grown in continuous darkness and microgreens (mainly root, hypocotyl, cotyledons, or young true leaves) grown under long photoperiod conditions. Previously, Lee and Cho, 2021 reported rosmarinic acid was present in roots (103.6 ± 5.6 μg/g), main-stems (70.0 ± 3.1 μg/g), sub-stems (116.6 ± 8.6 μg/g), leaves (84.2 ± 4.2 μg/g), and seeds (4606.4 ± 284.5 μg/g) of *P*. *frutescens* [5] and Kim et al., 2021 reported that *P*. *frutescens* leaves and stems produced rosmarinic acid, ranging from 5.44 mg/g to 20.603 mg/g and from 0.133 mg/g to 0.422 mg/g, during the vegetative stage of *P*. *frutescens* and showed that gradual decreases in rosmarinic acid in *P*. *frutescens* leaves and stems occurred during the reproductive stage of *P*. *frutescens*. In addition, spouts produced rosmarinic acid, ranging from 11.084 mg/g to 18.220 mg/g, during 16 days of long photoperiod conditions [27]. Therefore, this study suggests that *P*. *frutescens* microgreens grown under long photoperiod and continuous darkness conditions can produce high amounts of rosmarinic acid, comparable to adult plants. Furthermore, the DPPH inhibition patterns were consistent with the accumulation patterns of rosmarinic acid and total phenolics in both microgreens. This may be attributed to the presence of phenolic compounds, which are well-known for their strong antioxidant activities. Rosmarinic acid is a strong antioxidant [28].

*P*. *frutescens* microgreens exhibited antibacterial activity against *B*. *cereus* (KCTC 3624), *E*. *coli* (KCTC 1682), *P*. *aeruginosa* (KCCM 11803), *S*. *aureus* (KCTC 3881), *V*. *parahaemolyticus* (KCTC 2471), and *P*. *aeruginosa* (1113). These findings were consistent with those of previous studies reporting that extracts of different plant parts of various *P*. *frutescens* varieties showed antibacterial effects. For example, leaf essential oil and leaf extracts of *P*. *frutescens* var. *japonica* exhibited antibacterial effects against *B*. *cereus* (KCCM 11341), (KCCM 11774), and (KCCM 12667) [29]; *S*. *aureus* and *E*. *coli* [30], and leaf extracts, leaf essential oil, and shoot (stem and leaf) extracts of *P*. *frutescens* var. *acuta* showed antibacterial effects against *S*. *aureus* (ATCC 6538) [31], *E*. *coli* (ATCC 23736)*, P*. *aeruginosa* (ATCC 29336), *S*. *aureus* (ATCC 6538) [32], and *V*. *parahaemolyticus* (CCARM 7001) and (KCCM 41164) [33]. Furthermore, even though the total phenolic content and rosmarinic acid levels were not significantly different between microgreens under light treatment for 20 days and under dark treatment for 20 days, the light-treated microgreens showed higher antibacterial effects against the pathogens tested. This may have been due to the production of terpenoids. Terpenoid biosynthesis begins with the generation of glyceraldehyde-3-phopahte (G3P), which is derived from the photosynthetic carbon reduction cycle of photosynthesis, and acetyl-CoA, which originates from glycolysis. Therefore, photosynthesis is very important for this metabolism as it provides the most initial precursors. Terpenes have been reported to have strong antimicrobial effects [34], and *P*. *frutescens* essential oil contains various monoterpenes, sesquiterpenes, and antibacterial effects [35]. Therefore, this study suggested that microgreens under a long-day photoperiod could produce high levels of terpenoids, including carotenoids, via normal photosynthesis and stronger antibacterial effects than microgreens under continuous darkness treatment.

## 4. Materials and Methods

### 4.1. P. frutescens Microgreen Production

*P*. *frutescens* seeds were purchased from Aramseed Co. (Seoul, Republic of Korea). Five hundred seeds were located on each pot containing the vermiculites, and a total of 12 pots for whole dark treatment and 12 pots under cool white fluorescent light (Namyung Lighting Co., Seoul, Republic of Korea) with 20 µmol photons m^−2^ s^−1^ for long-day photoperiod (light/dark, 16/8) conditions were prepared. One pot containing 500 seeds represented one biological replicate, and three biological replicates were used in this study. After 10, 15, 20, and 25 days, microgreens grown in the dark and under light/dark conditions were harvested and washed with tap water. The microorganisms were stored at −80 °C, followed by freeze-drying. Dried microgreens were ground to a fine powder for further analysis.

### 4.2. High-Performance Liquid Chromatography and Determination of Total Phenolic Content

Rosmarinic acid and total phenolic contents in *P*. *frutescens* microgreenery grown under long-day photoperiod or continuous darkness conditions for 10, 15, 20, and 25 days were analyzed as previously described [36,37]. The dried powder (50 mg) of *P*. *frutescens* microgreens was soaked in 2 mL of 80% methanol, sonicated for 60 min, and centrifuged at 4000× *g* for 20 min. After filtration of the supernatant liquid, rosmarinic acid in the microgreen samples was quantified using an NS-4000 HPLC system (Futecs Co., Daejeon, Republic of Korea) as previously described [36,37]. To determine the total phenolic content, the dried powder (50 mg) of *P*. *frutescens* microgreens was mixed with 1 mL of 80% ethanol and then centrifuged at 4000× *g* for 20 min. The supernatant was then syringe filtered. Subsequently, 0.1 mL of each extract from the *P*. *frutescens* microgreens, 0.1 mL of each extract from the *P*. *frutescens* microgreens, 3.4 mL of distilled water, and 0.5 mL of Folin and Ciocalteu phenol reagent (2N) were added and incubated for 5 min. To stop the reaction, 2 mL of sodium carbonate (20% *w*/*v*) was added to the mixture, followed by incubation in the dark for 60 min and absorbance measurement at 760 nm using a spectrophotometer. Total phenolic content was expressed as gallic acid equivalents.

### 4.3. In Vitro Antioxidant Activity

2,2-Diphenyl-1-picrylhydrazyl (DPPH) scavenging activity of extracts from *P*. *frutescens* microgreenery grown under a long-day photoperiod or continuous darkness conditions for 10, 15, 20, and 25 days was assessed using a previously reported method [38]. A total of 1 mL of 0.3 mM DPPH ethanol solution and 0.1 mL extracts were placed into a cuvette and incubated for 15 min in the dark, followed by absorbance determination at 517 nm.

### 4.4. Screening for Antibacterial Activity

Antimicrobial screening of *P*. *frutescens* microgreens grown under a long-day photoperiod for 20 days or continuous darkness conditions for 20 days was carried out using a disk diffusion method [39]. Methanol (30 mL) was added to a flask containing *P*. *frutescens* microgreen powder (300 mg), and the mixture was shaken at 100 rpm for 24 h. Subsequently, the crude extracts were filtered through filter paper, followed by evaporation in a rotary vacuum evaporator. The evaporated extract was dissolved with DMSO and a final concentration of 50 mg/mL. For the bacterial cultures, *Bacillus cereus* (KCTC 3624), *Escherichia coli* (KCTC 1682), *Pseudomonas aeruginosa* (KCCM 11803), *Staphylococcus aureus* (KCTC 3881), *Vibrio parahaemolyticus* (KCTC 2471), *Salmonella paratyphi* C (KCCM 41577), *Streptococcus mutans* (KCTC 3065), *Micrococcus luteus* (KCTC 3063), and *P*. *aeruginosa* (1113) were briefly cultured to OD_600_ = 1.0 in each media. Next, a warm agar medium containing an aliquot (100 μL) of each bacterial culture was poured into a petri dish, followed by solidification. Two sterilized paper discs were placed on agar plates. Each disc contained 6 mg of extract of *P*. *frutescens* microgreenery grown under a long-day photoperiod for 20 days or continuous darkness. Each plate was incubated for 24 h, and the inhibition zones were measured.

### 4.5. Statistical Analysis

Duncan’s multiple range test was performed for all data from Table 1, Table 2, Table 3 and Table 4 using the SAS software (version 9.4, 2013; SAS Institute, Inc., Cary, NC, USA).

### 4.6. Chemicals

Folin and Ciocalteu′s phenol reagent, 2,2-Diphenyl-1-picrylhydrazyl, rosmarinic acid, and gallic acid and sodium carbonate and methanol were purchased from Sigma-Aldrich Korea (Yongin, Republic of Korea) and Samchun Pure Chemical Co., Ltd. (Pyeongtaek, Republic of Korea), respectively.

## 5. Conclusions

This study aimed to optimize the cultivation conditions (sequential darkness and long-photoperiod) for biomass productivity, rosmarinic acid production, and its biological activities in *P*. *frutescens* microgreens. Considering the dry weight, rosmarinic acid, TPC, and DPPH assays, *P*. *frutescens* microgreens after 20 days of darkness and 20 days of light treatment showed optimal conditions. Furthermore, methanolic extracts of both microgreens under light and dark conditions for 20 days showed strong antibacterial activity against *B. cereus* (KCTC 3624), *E. coli* (KCTC 1682), *P. aeruginosa* (KCCM 11803), *S. aureus* (KCTC 3881), *V. parahaemolyticus* (KCTC 2471), and *P. aeruginosa* (1113). To our knowledge, there are few studies on the antibacterial effect of *P*. *frutescens* microgreens against these pathogens and multi-drug-resistant pathogens. Therefore, these results suggest that *P*. *frutescens* microgreens can be a good source of rosmarinic acid production, as the optimal conditions for microgreen production were 20 days of long-photoperiod and sequential darkness treatment. Furthermore, the light treatment was considered the best condition for *P*. *frutescens* microgreen production due to its higher antibacterial effects.

## Figures and Tables

**Table 1:** The effect of light and dark treatment on dry weight of *P*. *frutescens* microgreens.

Exposure Time (d)	Dry Weight (g)	
	Light	Dark
10 days	0.51 ± 0.05 c ^1^	0.49 ± 0.18 b
15 days	0.86 ± 0.08 b	0.71 ± 0.05 ab
20 days	1.01 ± 0.10 a	0.90 ± 0.16 a
25 days	0.99 ± 0.05 ab	0.87 ± 0.12 a

^1^ Means with the same letter are not significantly different at *p* < 0.05 using DMRT.

**Table 2:** Production of rosmarinic acid in *P*. *frutescens* microgreens grown under long-day photoperiod or continuous darkness conditions.

Treatment	Duration	Rosmarinic Acid (mg/g Dry Weight)
Dark	10 days	9.01 ± 7.36 b ^1^
	15 days	13.01 ± 2.82 b
	20 days	22.66 ± 0.55 a
	25 days	13.75 ± 20.68 b
Light	10 days	21.89 ± 0.80 a
	15 days	20.18 ± 1.40 a
	20 days	21.08 ± 1.20 a
	25 days	20.68 ± 2.93 a

^1^ Means with the same letter are not significantly different at *p* < 0.05 using DMRT.

**Table 3:** TPC and DPPH assay of *P*. *frutescens* microgreens grown under long-day photoperiod or continuous darkness conditions.

	Light				Dark			
	10 Days	15 Days	20 Days	25 Days	10 Days	15 Days	20 Days	25 Days
Total phenolics [mg gallic acid equivalent (GAE)/g Dry weight]	2.52 ± 0.23 a ^1^	2.43 ± 0.04 a	2.59 ± 0.24 a	2.64 ± 0.37 a	1.08 ± 0.59 b	1.49 ± 0.16 ab	2.64 ± 0.77 a	1.39 ± 0.80 b
DPPH (inhibition%)	95.97 ± 0.04 a	95.54 ± 0.14 ab	95.32 ± 0.47 b	95.37 ± 0.39 ab	94.51 ± 0.32 c	95.09 ± 0.21 b	95.87 ± 0.25 a	94.43 ± 0.07 c

^1^ Means with the same letter are not significantly different at *p* < 0.05 using DMRT.

**Table 4:** Antibacterial activities of methanol extracts of *P*. *frutescens* microgreens for 20 days of long-day photoperiod and continuous darkness conditions.

Group	Bacterial Strains	Zone of Inhibition (mm)	
		Extracts from *P*. *frutescens* Microgreens after 20 Days of Light Exposure	Extracts from *P*. *frutescens* Microgreens after 20 Days of Dark Exposure
Pathogens	*B*. *cereus* (KCTC 3624)	12–13	13–14
	*E*. *coli* (KCTC 1682)	10–11	13–14
	*P*. *aeruginosa* (KCCM 11803)	13–14	17–18
	*S*. *aureus* (KCTC 3881)	11–12	12–13
	*V*. *parahaemolyticus* (KCTC 2471)	10–11	12–13
	*S*. *paratyphi* C (KCCM 41577)	─ ^1^	─
	*S*. *mutans* (KCTC 3065)	─	─
	*M*. *luteus* (KCTC 3063)	─	─
Multidrug resistant pathogens	*P*. *aeruginosa* (1113)	11–12	13–14

^1^ negative.

## Data Availability

Not applicable.
